# The harms of medicalisation: intersex, loneliness and abandonment

**DOI:** 10.1177/14647001211062740

**Published:** 2022-02-20

**Authors:** Charlotte Jones

**Affiliations:** 3286 University of Exeter, UK

**Keywords:** Community, disorders of sex development, feminism, intersex, loneliness, medicalisation, relationships, shame, variations in sex characteristics

## Abstract

This article develops loneliness as a political and social justice issue by illustrating the harmful personal and social consequences of the medical jurisdiction over and constitution of variations in sex characteristics. Whilst connections between loneliness, health and illness have been well established, this work customarily identifies the ways illness can lead to, or be caused by, loneliness. Instead, I provide an account of the central role of medicalisation and medical management in producing loneliness. By doing so, I underline the imperative for medical practice to consider its influence upon social and personal, as well as physical, wellbeing. Drawing on stories shared through solicited diaries followed by in-depth interviews with seven people with sex variations and two parents in the UK, I show how accounts of loneliness help to illuminate the violence of abandonment, silencing and marginalisation that often goes unheard, together with hidden or normalised systems of harm.

Building on concepts of ethical loneliness and ontological loneliness, I show how structural violations operate to injure trust and self-worth, leading to social unease. I argue for the importance of people with sex variations finding sites of comfort and acceptance, but note the ways that some forms of medicalisation can inhibit alliances and community formation, despite diagnoses also carrying the potential to facilitate informal support structures and collective identities. By bringing together intersex studies with discourses of loneliness, I develop a better understanding of loneliness as a product of social and systemic violence, and the ways in which medical discourses tie in with larger structures of oppression, coercion and control. This article concludes by underlining the need for structural change in our approach to and understanding of sex variations, and with a call for us to become more attentive to these stories of medical harm, to ensure that they are heard and to seek necessary justice.

## Introduction

Ways of thinking about loneliness often obfuscate the conditions of social power in which the feeling occurs. This article seeks to explore loneliness as a product of ‘violence and harm’ (De Bie, 2019: 1166) that may result from the social abandonment and marginalisation of subjects or groups (Stauffer, 2015). Whether through omission or contempt, some lives are made to feel less valuable than others, rendered both as ‘invisible and hypervisible’ (Guenther, 2013). Solace or resolution is not always found in our relationships with others, and sometimes these relationships only heighten a feeling of distance.

This article addresses the ways in which people with variations in sex characteristics (VSCs), or intersex traits, experience injustice through processes of medicalisation and ‘compulsory dyadism’ (Orr, 2018, 2019). Seeking to understand these harms as both systemic and socio-political, I move beyond models of loneliness in which the individual is seen as responsible for either the problem or the solution, instead implicating pernicious and binding discourses and structures of power, and expanding on wider social justice concerns regarding the mistreatment of people with VSCs. To do so, I explore loneliness in two forms: ethical loneliness (Stauffer, 2015), which may follow when a significant trauma or injustice has not been socially heard or recognised, causing a loss of trust in the belief that others will treat you well, or that they will recognise the significance or actuality of the harms to which you have been subjected; and what I term ‘ontological loneliness’ (Jones, 2016: 68-69), whereby the interconnectivity required for identity- and self-formation may be denied, and a coherent and cogent conception of self may feel out of reach. I argue here that these feelings of loneliness are produced by processes of over-medicalisation and unnecessary medical practice, resulting in the disavowal of the status, bodies and futures of people with sex variations, whereby limitations are placed on their ways of being and knowing themselves.

This article's reach extends beyond the field of intersex/VSCs, as concerns with loneliness are fundamental to a breadth of sociological theory. I illustrate how the personal and private experiences of loneliness can be interpreted as public, structural and social concerns, providing new insight into the important connections between social relationships, perceptions of self and the impact and power of wider social structures. This work is also situated alongside other valuable feminist research taking a critical approach to concepts of sex, epistemic authority and essentialist and binary paradigms. People with sex variations are often subject to significant sex- and gender-based discrimination (or interphobia), and it is vital for feminist scholarship to recognise this alongside and within other forms of sexist, queerphobic and ableist violence and oppression.

## Loneliness

According to Mills ([1959] 1978: 14), the relationship between ‘personal troubles’ and ‘the public issues of social structure’ should be central to all social theory. The fraught, complex and heterogeneous interconnection between the individual and society, and the constitution of these two concepts, is fundamental to much of our thinking on loneliness, its causes and its significance. Even when loneliness is not wilfully addressed, it lies beneath the surface of a range of work on social relationships, as well as the ontological and epistemological value and security of ‘the individual’. Most fundamentally, this is illustrated by the common belief that our potential, pleasure, worth and even coherence as individuals are recognised only in and through our meaningful relationships and interactions with others.

Developing an understanding of the emotional and social struggles of people with variations in sex characteristics requires insight into the fundamental and interdependent relationships between the constitution of the personal, social and structural facets of our lives. This includes a recognition of how social structures can influence, or determine, individual behaviours and choices; that personal actions can never be performed or interpreted outside of a social context; and that the actions of (those with) state and institutional power have crucial, but perhaps unnoticed, effects upon others. Thus, there is nothing ‘natural’ or ‘inevitable’ about these processes or structures, or about loneliness as a symptom or consequence. Loneliness is also not typically determined by the number of a person's social connections. Regardless of the multifarious friendships, family and other relationships one may have formed, loneliness can nevertheless develop and persist if social needs do not feel fulfilled or satisfied entirely (Rokach, 2014), and existing bonds are perceived as insufficient or perfunctory (Weiss, 1973; Peplau and Perlman, 1982; Hortulanus et al., 2006). ‘Random sociability’ (Weiss, 1973: 17) may in fact aggravate feelings of loneliness rather than alleviate them. Indeed, a sense of loneliness can also lead to further loneliness. The social value placed upon our social connections – especially the pressure to form what is considered to be ‘meaningful’ or ‘intimate’ relationships – may result in feelings of shame and humiliation when one is perceived to be unsuccessful. This, in turn, can compound isolation or feelings of inadequacy (Seabrook, 1973: 9). That is, when social bonds are framed as fundamental to physical and mental health, and to a high quality and ‘successful’ life, those who are outcast may be disavowed even more severely, and their sense of being – without approval, recognition and engagement from others – may cease to ‘make sense’.

For centuries, scholars have recognised loneliness as a pervasive problem, framed as both an existential driving force behind all human behaviour (Mijuskovic, 2012) and a crucial part of all lives (Peplau and Perlman, 1982). Its alleged ubiquity has led to a turn towards the ‘the ordinary loneliness of ordinary people’ (Weiss, 1973: 9; see also: Seabrook, 1973; Hortulanus et al., 2006), and a push for a better understanding of mundane and commonplace feelings of loneliness, rather than only what may be recognised as severe marginalisation in extraordinary or unusual situations. The uniformity of loneliness, however, has not diminished its perceived threat. Concerns have been raised about the dangers of loneliness at a national level, with Britain declared ‘the loneliness capital of Europe’ (Bingham, 2014) and the current moment defined as an unprecedented ‘Age of Loneliness’ (Monbiot, 2014; see also: Alberti, 2019; Cooper, 2020; Vincent, 2020). Whilst new societal-level loneliness ‘crises’ are announced periodically (Cooper, 2020), loneliness discourses are too often individualised and pathologised, placing a responsibility to be more resilient on individuals and communities who are struggling (Stenning and Hall, 2018; Duggan, 2020), with little attention given to a broader landscape of inequality and disenfranchisement (Cooper and Jones, 2021).

Sociopolitical explanations for the alleged loneliness crisis, on the other hand, reach beyond personal expressions of isolation, instead considering social, structural and institutional factors which produce – or make more probable – loneliness at this particular moment. In recent decades, these arguments have principally focused on individualistic and neoliberal ideologies as a leading cause (e.g. Putnam, 2000; Franklin, 2009; Monbiot, 2014), whereby a Western valorisation of hedonistic and competitive attitudes is said to have resulted in alienation, emotional distancing and isolation, and where austerity measures have eroded public facilities, community centres and support services (Stenning and Hall, 2018). Further, through this model, loneliness is not (only) an affective state felt by individuals, but also a collective response to mistreatment and disparity, whether through abandonment, disregard, contempt or otherwise. On these terms, loneliness is rightly understood as a social justice issue. Developing this line of analysis, I turn to other harmful structures – namely coercive medical and social systems of sex binarism – to consider the critical role played by our social environments and infrastructures in producing the possibility of emotional distress and loneliness. Accounts of loneliness help to illuminate the violence of neglect, silencing and marginalisation that often goes unheard, together with hidden or normalised systems of harm. Establishing a structural model of loneliness not only helps us to think differently about the concept and its causes, but also moves us away from individualistic and pathologising solutions.

## Mistreatment of sex variations

Over the last thirty years, intersex scholarship and activist discourses have emphasised ethical concerns with the pervasive medical approach to variations in sex characteristics, which assumes that early intervention is necessary. Whilst some significant changes to medical guidelines have been developed over the last two decades (e.g. Lee et al., 2006; Lee et al., 2016), including a more psychosocial approach, intersex births are still persistently described and perceived as a ‘medical and social emergency’ (Özbey et al., 2004: 388; see also: Garland and Travis, 2020a). Within medical protocol, ‘corrective’ surgery and other interventions portrayed as serving the purpose of ‘normalisation’ are often performed before the child is of an age to consent or fully understand procedures, with lasting impact on fertility and the sensation, function and appearance of genitals, often leading to follow-up treatments in youth and adulthood (Karkazis, 2008). For many, these irreversible interventions illustrate the way in which intersex bodies are understood to be ‘unacceptable, perhaps unlovable, and certainly unrecognisable as persons’ (Holmes, 2008: 170).

Conversely, early medical procedures are often framed by healthcare professionals as a means to prevent social stigma and isolation, and to support psychosocial wellbeing (e.g. Money, 1994; Meyer-Bahlburg, 2008; see also: Liao et al., 2019). This is due to a perceived (and contested) improvement to genital appearance and body image, thus potentially avoiding social ostracism and supporting adult sexual intimacy. However, many adults have reflected on their experiences as painful, deeply traumatic and distressing, leaving them anxious about ‘abnormalities’ (Davis, 2015). They have also compared interventions and examinations during childhood and youth to rape and sexual abuse (e.g. Kessler, 1998; Preves, 2003; Ehrenreich and Barr, 2005; Tosh, 2013). These psychological and emotional consequences, as well as the physical harms caused by intersex medicalisation and ethical violations, have been widely attested in the literature (e.g. Preves, 2003; Holmes, 2008; Karkazis, 2008; Davis, 2015). Chase concludes that ‘the problem’ of sex variation is ‘stigma and emotional trauma’; current medical practice, she notes, produces patients who feel ‘utterly unique, alone, and unacceptable’ (2003: 240).

The high level of medical scrutiny and subjection to non-consensual practices has brought other sustained and far-reaching consequences which can result in loneliness and isolation. Some adults with sex variations describe distrust in the medical profession (Roen, 2008; Davis, 2015), a compromised sense of autonomy or control over their lives and bodies (Preves, 2003; Feder, 2014; Jones, 2016), a lasting feeling of indignity (Tosh, 2013), an unease with and avoidance of sexual intimacy (Davis, 2015; Jones, 2016), a feeling of fraudulence, and a perception of doubt or suspicion from others (Jones, 2016). Some research highlights a strong disconnect between how clinicians and people with sex variations view current healthcare provision (Jones, 2018), and there have been calls for a more patient-centred model, with the intention to ‘empower’ and prioritise the individual patient's goals (Jones, 2018). In the case of early diagnoses, the pivotal role parents take in making decisions about their children's wellbeing and medical treatment also has fundamental ramifications, including parental isolation (Lee et al., 2016) and guilt (Davis, 2015: 116), which could in turn lead to further difficulties for children (Chivers et al., 2017).

Due to shame, stigma and imposed secrecy (Preves, 2003; Karkazis, 2008; Feder, 2011, 2014; Davis, 2015), people with VSCs often feel unable to speak to others about their sex/variation or their medical treatments (Liao et al., 2010; Jones, 2016; Jones, 2020; Schweizer et al., 2017) and therefore feel as though they are being dishonest or ‘hiding’ details of their lives (Jones, 2016). Stigma, rejection and discrimination have also led to increased levels of anxiety, depression and social isolation amongst people with sex variations (Zeeman and Aranda, 2020). All these factors can act as significant barriers to accessing health and social care services (Zeeman and Aranda, 2020). Shame itself can also endanger relationships and feelings of belonging (Dolezal, 2015). Loneliness scholars often underline the significance of forming intimate, open and ‘confiding’ relationships as a departure from loneliness (e.g. Weiss, 1973; Brown and Harris, 1978; Lynch, [1977] 1979; Franklin, 2009), with the assumption that this intimacy is most likely found in romantic partnerships. Whilst the centring of romantic relationships could be questioned, this common perception also poses difficulties for some people with sex variations, who may not find these connections easy or comfortable. Further, complete transparency may hold greater risks for people with variations in sex characteristics, complicating the desirability of a confiding relationship.

A recent shift in medical nomenclature has also led to greater feelings of marginalisation amongst some people with VSCs (Davis, 2015). All sex characteristics which do not fit discrete medical expectations of male or female criteria – affecting chromosomes, genetics, hormones, secondary sex characteristics, reproductive organs/gonads or genitals (referred to here as VSCs) – are now clinically termed ‘disorders of sex development’ (DSDs) (American Psychiatric Association, DSM-5 Task Force, 2013). This label and classification system has been profoundly contentious within intersex/VSC advocacy movements (see: Davis, 2015), with concerns largely lying in its potential to position sex variations semiotically within the biomedical paradigm, heightening pathologisation and passivisation, whilst furthering the ‘requirement for the unexpected body to be rectified’ (Holmes, 2011: 395). This also brings potential legal consequences, hindering pursuits for recognition and protection for people with VSCs (Creighton et al., 2009: 259), especially when ‘legislatures rarely interfere with medical protocols’ (see also: Garland and Travis, 2020b). The language of ‘disorder’ is understood not only to medicalise bodily diversity but, in doing so, to denote abnormality (see: Foucault, 1980).

The DSD diagnostic taxonomy is the most recent medical classification system in a historical succession of attempts to define and clinically manage VSCs (Griffiths, 2018). People inside and outside the medical profession, including intersex activists and people with VSCs, have different views on what should be included within these classifications, and biotechnological changes have also transformed current approaches and future possibilities for defining, detecting and predicting physical differences (Griffiths, 2018). Delimata (2019) has shown how disagreements over terminology are indicative of a clash between two discordant ontological perspectives: the empirical corporeal knowledge of the reality of sex diversity on the one hand, and the social ideal that sex is binary on the other. These differing ontologies result in contrasting interpretations of VSCs, their meaning and management. DSD terminology (and the broader medicalisation of VSCs) thus positions sex variations as an individual's ‘problem’ which healthcare can help to resolve.

Feminist scholars writing on intersex politics have argued that the institutional treatment of VSCs holds particular relevance to feminist discourses. Traditional medical models of sex have been identified as a way to bolster a heterosexual imperative (Kessler, 1990; Butler, 1993; Fausto-Sterling, 1993; Feder, 2009; Davis, 2015) and perpetuate misogynistic and patriarchal values (Holmes, 1995; Chase, 1998). Accounts from VSC medical specialists have also brought to light the heterosexist beliefs held by doctors regarding gendered behavioural and physical expectations of children (Kessler, 1990; Karkazis, 2008; Feder, 2009; Davis, 2015). Similarly, Holmes (1995) has critiqued the gender regulation employed in early genital surgeries, which are founded upon the extent to which the appearance of the genitalia transgresses norms for a particular sex. Chase (1998: 207) considers the medical approach to intersex to be of particular detriment to women. She describes medical intervention as ‘another form of violence based on a sexist devaluing of female pain and female sexuality’ (Chase, 1998: 207), whereby, she notes, a girl growing up without a clitoris or ovaries is favoured over the prospect of a boy with a small penis. More broadly, intersex scholarship is concerned with how biomedical models, as well as geopolitical and social histories, inform our understanding of gender, bodies and classification frameworks, and bring various disciplinary, normative and ethical implications (Butler, 1993, 2004). For these reasons, it is essential for intersex research to both draw from, and contribute to, feminist theory.

Various scholars of loneliness have pointed to the complex relationship between health, social and personal needs (e.g. Lynch, [1977] 1979; Elias, [1985] 2001; Rosedale, 2009), and loneliness itself has increasingly been positioned as a public health crisis. Indeed, a range of quantitative empirical studies evidence how loneliness can affect health, including its potential impact upon blood pressure (Hawkley et al., 2010), and an increased risk of coronary heart disease and stroke (Valtorta et al., 2016). These health consequences are often outlined as a way of substantiating the collective urgency to end loneliness, although doing so may discount the emotional significance of, and damage done by, loneliness itself. Conversely, it has been illustrated that disability, mental/physical illness and chronic conditions – or the associated ableism – can also lead to experiences of loneliness (e.g. Charmaz, 1983; Elias, [1985] 2001; Rosedale, 2009; De Bie, 2019). There is a lack of understanding, however, of the ways in which medicalisation, medical management and the contestation of a diagnostic status can themselves play a central role in producing loneliness. The biophysical is at once a somatic and social state, in part ‘created and shaped by human knowledge and evaluation’ (Freidson, 1970: 223). Thus, in line with previous sociological health literatures, this article underlines the imperative for medical practice to consider its influence upon social and personal, as well as physical, wellbeing.

By bringing together intersex studies with discourses of loneliness, I seek to develop a better understanding of loneliness as a product of social and systemic violence, and the ways in which medical discourses tie in with larger structures of oppression, control and abandonment (Foucault, 1980; Butler, 1993; Fausto-Sterling, 2003). The narratives of people with VSCs illustrate the deeply personal level at which the ideologies and policies of the state are felt and understood, whilst nevertheless also demonstrating their persistence in seeking and nurturing communities and solidarities. The complex, disparate and unique histories of medical and social harms encountered by people with VSCs are too often neglected, in part due to the jurisdiction of medicine to ‘transform if not actually create the substance of its own work’ (Freidson, 1970: xvii), thus rendering social critiques about mistreatment invisible. A note on terminology before I reflect on methodology below: in this article, the terms ‘variations in sex characteristics’ (VSCs) and ‘sex variations’ are used most often, due to their utility and translatability across a range of viewpoints. Other terms, such as ‘intersex traits’, ‘disorders/differences of sex development’ (DSDs) and specific diagnostic labels are also used in the article according to the context, the terminology used or preferred by participants or the particular literatures discussed.

## Research design

The themes developed in the discussion below arose from a small qualitative study conducted between October 2013 and October 2014 (Jones, 2016, 2020, 2022). The research aimed to explore the social and medical experiences of people with VSCs or intersex traits in the United Kingdom (UK), focusing in particular on medical diagnosis and its influence upon / interaction with the realisation of identities and interpersonal relationships. This involved a two-tiered process of solicited diaries followed by in-depth unstructured interviews (Zimmerman and Wieder, 1977) with five women and two men with VSCs, and two parents of children with VSCs. The nine participants initially wrote about their experiences in unstructured, reflective diaries over a period of two months, then the same cohort attended in-depth interviews to build on the themes observed in the diaries (Jones, 2022). The full data set provides the basis for this article. Interviews were one-to-one, in-person, and took place in areas local to participants. A short list of key themes informed all interviews, together with each participant's diary content, which was used as a primary resource for shaping their respective meetings. Questions were unscripted, and the interviews were informal and partially participant-led in order to share some control over the direction of the conversations.

All nine participants were white, aged between twenty-two and fifty-four and from diverse class and educational backgrounds, with a range of sexualities. They had been given a variety of diagnoses: Turner syndrome, complete and partial androgen insensitivity syndrome, hypogonadism, congenital adrenal hyperplasia and Swyer syndrome. Participants were recruited through a range of online sources: internet forums for intersex people, and support group emailing lists; websites; and Facebook groups designed for the discussion of intersex or variation-specific issues. The participants’ experiences of race and racialisation, and its intersections with other social axes, such as class, age, sexuality, nationality, gender and VSC, will have inevitably had an impact on their stories, identities and outlooks, as well as their experiences of marginalisation, discrimination and loneliness. Whilst the small size of the research project and the space constraints of this article place limitations on its breadth of analysis, there is a vital need for more detailed intersectional accounts of intersex loneliness, particularly work foregrounding race and racism, which are currently under-explored in intersex studies.

Diaries and interview transcripts were categorised into a coding scheme and separated thematically. This was followed by content analysis (Holsti, 1969) to systematically identify key features and themes. The interviews and diaries included a range of topics, but loneliness emerged quickly as a core ‘bridging’ focus of the work due to its salience across all accounts and analytic sub-categories (see also: Jones, 2016). In the following sections, I develop the role and production of loneliness for people with variations in sex characteristics across five key themes: shame, value, community, belonging and difference. These themes elucidate the ways in which medical jurisdiction over – and constitution of – sex variations have harmful personal and social consequences. By drawing on accounts of ethical and ontological loneliness, I illustrate how structural violations operate to injure self-worth and trust in others, leading to social unease, isolation and alienation. I argue for the importance of making sites of comfort and acceptance available, and note the ways that medicalisation potentially inhibits alliances and community formation. This article concludes by underlining the need for structural and social change in our approach to and understanding of sex variations, and with a call for us to become more attentive to these stories to ensure that they are heard.

## Shame

Shame emerged as a powerful impulsion in participants’ narratives, particularly personal shame about the appearance and meaning given to their own bodies, but also shame about others’ interpretations of their bodies or VSC diagnoses. After spending the Christmas break with family, Pandora told her parents that she needed to return to university to study. Instead, she travelled to the hospital to undergo vagina surgery alone. In her diary, Pandora's memory of this time is distinctly solitary, and magnified by the illusory visions she experienced of a well-populated hospital ward. She wrote of the confusion she felt after waking up from surgery and starting a programme of opioid analgesic drugs: ‘I didn’t hallucinate as such, but each time I closed my eyes I microdreamt that I was surrounded by people, only to open my eyes to an empty room’. Without informing any of her friends or family, at nineteen years old Pandora sustained surgical treatment to extend her vagina cavity. She chose to undergo a technique similar to vaginal dilation, the Vecchietti procedure, which is a one-step medical intervention in which the vagina ‘dimple’ undergoes continuous pressure for between seven and ten days, in order to enlarge the cavity. Pandora was in hospital under supervision for the duration of the procedure, undergoing a tightening of the medical apparatus each day, which caused a ‘huge amount of pain’ and meant that she was unable to stand independently during the course of treatment. Pandora lost ten pounds in weight during her stay and, once the apparatus was removed, she was still unable to walk for a further two days.

Pandora, twenty-two years old at the time of interview, was given a diagnosis of complete androgen insensitivity syndrome (CAIS) at six months old, but she was not informed of this until her mum shared the diagnosis with her when she was eleven. Not only did Pandora find her sex variation and her ‘non-conforming’ body to be shameful, but she was also deeply embarrassed by the thought of undergoing surgery. The shame she experienced was so acute that it felt crucial that the surgery was kept entirely confidential, leading Pandora to orchestrate her own isolation during this time. She needed to withstand the operation on her own, in secret. The isolation Pandora described over this period was not due to an absence or lack of close relationships (see: Peplau and Perlman, 1982). Rather – due to circumstances in which Pandora was ashamed and stigmatised by her body, diagnosis and treatment – she felt restricted in her ability to share her struggles or look to others for support and companionship (see: Dolezal, 2015).

Pandora's sense of shame gave rise to other critical consequences; she spoke of suicidal intentions once the medical procedure had been undertaken. She explained in her diary:

the idea of the surgery was so shameful that I couldn’t live with it. So I would have the surgery and take my life after. I prepared, planned and obsessed. It was an awful term at uni. Knowing I was going to die made everything so futile; I was reclusive and couldn’t sleep. My work suffered, as did my friendships.

In anticipation of her surgery, Pandora described an increasing detachment from society. Whilst much of this seclusion was framed as self-imposed, the shame and associated feelings of purposelessness and futility she experienced reflected her judgment of her own social value. The loneliness and suicidal intentions Pandora recounted were not simply individual expressions of distress, but – as Durkheim ([1897] 1951) argued – responses to structural constraints and harmful systems. The medicalisation of sex variations is consistent with a wider social and systemic valorisation of naturalness, bodily integrity and ‘compulsory dyadism’ (Orr, 2018, 2019). Pandora's perceived failure to be the person she felt she needed – or was urged – to be led to her feelings of deficiency and embarrassment and a self-imposed social isolation (see: Dolezal, 2015; Ypsilanti et al., 2019). When we ‘take loneliness seriously as a form of violence and harm’, De Bie describes, ‘it emerges as a sense of lostness, loss, and being at a loss’ (2019: 1166; see also: Charmaz, 1983). Pandora's life was not worth living because she feared it was a life, and a body, which did not satisfy dominant paradigms of social value; she did not have a place here, she was at a loss.

### Value

Some participants expressed an acute sense of social rejection and abandonment. This meant that not only did they feel they lacked sufficient companionship but they also found it especially challenging to seek new friendships or support. Ian, forty-four years old, reflected on how the bullying he experienced during childhood impacted his current ambivalence towards forming new social connections:

I think because of all the ridicule as a child, I reacted with withdrawing and isolating myself. I think isolating myself, it led to some … difficulties, I think developmental problems, and I think the lack of friendships, relationships growing up, that led to … difficulty in social interactions. Then the experiences of people that were there, the ridicule and the rejection of me. And … you get to the frame of mind that, you know, people won’t accept me, so I tend to isolate myself. But I guess it's about trying to change my core beliefs about myself, that there are people out there who will be kind and positive. But yeah, I have a hard time with that, trying to engage with people.

Ian acknowledged that his negative experiences had led to a sceptical and cautious approach to forming new relationships. His self-imposed isolation acted as a security barrier, protecting him from further social harm, albeit with a cost of loneliness. Furthering his hopelessness, Ian regarded his recent attempts to form new friendships as similarly discouraging. After a colleague withdrew from their arranged social event, spreading disparaging rumours about Ian in the workplace, he concluded, ‘my perception was that, y’know, he didn’t want to be out with someone like me’.

Whilst Ian frequently reassured himself that there were ‘some people [who] may be more understanding and supportive’, he also normalised (and to some extent vindicated) others’ hostility by depicting their aversion as a symptom of universal and instinctive ‘human *fear* and disgust and hatred’. Ian could not rely on being treated well, and his ‘trust in the world’ as ‘[benevolent,] kind and caring’ (Stauffer, 2015: 78) had been destroyed. Thus, whilst he attempted to re-learn the valuable and enjoyable potential of friendship, he still battled with ‘a loss of safety’ (Stauffer, 2015: 27) and the perceived inevitability of social rejection. Ian also indicated that he was partially culpable for his neglect, remarking that, ‘I have to kind of get out there and be more open and less shameful’, whilst also excusing his experiences of physical and verbal assault, commenting that he ‘need[ed] to stand up for myself more’. By assuming responsibility, Ian felt penitent about his abandonment and marginalisation, a further injury to his self-worth.

In Pandora's diary, she detailed how feelings of inferiority led to her alienation during social engagements. Similar to Ian, Pandora felt a distrust in the value of her social contributions. She explained that ‘when the feeling of difference descends it's like a pane of glass has appeared between me and them, and although I’m a few feet away I feel so many miles away. And it's all because I don’t deserve to be a valued member of a group. Outsider in terms of identity, so outsider socially’. The feeling of difference, discussed later in the article, was described by Pandora as generating a sense of shame, inferiority and loneliness. She made an explicit connection between her intersex status and her social circumstances, indicating that the way in which her body confounded medical recognition led to her being socially omitted at an everyday, interactive level. Pandora's example also illustrates how loneliness can persevere despite the presence of others. Instead, this was a feeling rooted in her perceived lack of value. She explored this further in her diary, noting: ‘in groups, I often find myself as the bystander, there but not contributing, watching people laugh together but not laughing with them. For in my mind I am ranked below them, the whole, worthy, beautiful people. Nothing I can say is ever of worth, as it comes from this incomplete lesser’.

A sense of personal inadequacy can be coupled with experiences of loneliness (Brown and Harris, 1978; Peplau and Perlman, 1982; Pritchard and Yalch, 2009; Ypsilanti et al., 2019). In Pandora's account, her feelings of social incompetence and inferiority prohibited her from contributing to interactions in the way she felt she should. Pandora's sense that she was not adequately performing must have also been a burden for her. Fricker describes how, for those with less social power, collective experiences can be lived ‘through a glass darkly, with at best ill-fitting means to draw on in the effort to render them intelligible’ (2007: 148). For Fricker, this is an issue of hermeneutical marginalisation, whereby certain people experience a disadvantage of interpretation and participation, deprived both from self-understanding and from generating meaning from our social world. Pandora could not find a way to share or communally explore a world which did not seem to recognise her. Her loneliness was felt at an ontological level.

Pandora's reflections illustrate how different lives are tangled up in each other; in many ways dependent and vulnerable, with the knowledge that ‘the selves and worlds of human beings can be destroyed by other human beings’ (Stauffer, 2015: 167), but are also ‘built cooperatively, by human relationships’ (Stauffer, 2015: 80). Drawing on Althusser's concept of interpellation, Delimata (2019) describes the phenomenon of ‘disinterpellation’ trauma, which occurs due to an epistemic incoherence between the empirical reality of sex variation and the social idea of binary sex. In other words, due to presumption that sex is dualistic, Delimata explains, a VSC diagnosis from a trusted medical authority ‘hails the patient out of socially coherent existence’ (2019: 181–182). Any degree of control or autonomy one has over their presentation of self, however compromised, will still need participation to make it meaningful; we require others ‘to acknowledge its worth and thus observe its boundaries’ (Stauffer, 2015: 16). Hence, power differentials that structure the very making of our worlds and understanding of our bodies inevitably carve out injustice at an ontological level. The world itself is organised ‘by others for their purposes – purposes that at the very least are not our own and that are in various degrees inimical to our development and even existence’ (Hartsock, 1998: 241). As described in the section above, the distress and sense of incoherence which emerges here does not, as Delimata (2019: 184) also argues, result from the person's body, but rather from evaluative and incompatible ‘beliefs about how sexed bodies *ought* to be’ (see also: Freidson, 1970).

### Community

There was a persistent emphasis on the significance of a supportive and understanding community in the interviews, and the parents of children with VSCs repeatedly shared a fear of their children's impending isolation. They discussed a desire to ensure their daughters grew up knowing other people with the same sex variation, as they believed this was a way of helping to guarantee their children would have necessary support. Nicole, whose twenty-seven-year-old daughter was diagnosed with Turner syndrome a few days after birth, emphasised her parental duty to ensure her daughter felt socially accepted. In her interview, she reflected on the attention she has since given to campaigning about Turner syndrome, underlining the motivations behind her involvement: ‘I’m still just a *mum*. A mum that … wants a better quality of life for her daughter, and her not to feel isolated, and to feel any less a person because she's been given this diagnosis’.

Nicole's anxieties – attached to the medical diagnosis – foreshadowed her daughter's social existence, emerging when she was days old and long before she was able to express her own fears or struggles. To prevent these potential difficulties, Nicole worked hard to build a support network of people with similar circumstances to her daughter. The strong imperative to provide this was made especially clear when Nicole spoke of her concern for people living without this community. She explained, ‘the people that I *really* worry about are the people that have never met anybody, that have led these very isolated lives, that have not had the wonderful opportunities that [support group] members get, of being together’. Nicole characterised the support gained from interacting with others with the same diagnosis as different to other kinds of support:

There's a kind of magic that these girls get from each other. […] And they all help each other, and they’re all supportive of each other. And you still get the odd niggle, but on the whole they are incredibly nice, really nice – silly nice […] because the girls just *love* being together. And when you talk to the girls and you say, ‘what is it about you, why do you love being together?’ – they say ‘we can be ourselves, we don’t need to …’ because I think they spend their entire life trying to fit in. And lots of […] parents [of children with Turner syndrome] say that the girls are like round holes in square pegs. So they rattle about but they don’t *fit*. And only when they come to [the support group events] do they fit. But that has its problems. Because some of them, it's so important that they don’t want to leave.

Nicole recognised the support group environment as one that allowed its members to behave in a way that was not permitted in everyday social contexts, offering the security of a community. This was beneficial largely because she believed that people with a diagnosis of Turner syndrome felt excluded from conventional social settings, or were made to feel they could not ‘be themselves’. However, she also noted how these sites of comfort could present a challenging reminder to their members of how unsettled and laboured they found everyday experiences. For some, this effort – and, indeed, the contrast between safe and unsafe social environments – may have exacerbated feelings of ontological loneliness. Whilst Nicole's support group coalesced under diagnostic terminology and worked in collaboration with clinicians, her goals for the community were not explicitly curative, but instead based in social change, including the celebration, understanding and enablement of diversity. Variation-specific groups inhabit a complex relationship with medical frameworks, but can nevertheless grant the possibility of legitimising subjective and embodied experience (Brown and Zavestoski, 2004), and were described by some participants as their first opportunity to voice and hear criticisms of, and alternatives to, biomedical models or treatments.

In Rosedale's (2009) work with survivors of breast cancer, she illustrated the connections made between the sharing of medical experiences and the escape from or suspension of loneliness. Participants described ‘feeling lonely when they realised that others were not aware of an ongoing aspect of their breast cancer experience’ (Rosedale, 2009: 178), including the fear felt before an upcoming mammogram or the significance placed on the anniversary of their cancer diagnosis. Women in the study felt that ‘people failed to recognise and comprehend what it was like to survive acute treatment and the long-term aftermath of breast cancer, which led them to become conscious of their loneliness’ (Rosedale, 2009: 178). This bears a resemblance to Nicole's description of the dejection some members felt following support group meetings. Nicole reflected, ‘they go away and […] some of them struggle’ because ‘it's tough out there’ and ‘being different […] in this country, it is *not* celebrated’. In the case of sex variations – as Nicole underlined – the value of spending time with others who have shared similar experiences stretches beyond a matter of common ground and understanding, as these spaces may also be perceived as a refuge from discrimination, ostracism and hostility, and a liminal site whereby members may begin to explore different ways of conceptualising their bodies, diagnoses and communities.

### Belonging

Variation-specific and intersex support groups did not always provide a solution to social discomfort or loneliness for research participants, and the pursuit for community and belonging was presented as complex and compromised. Whilst most participants who made regular use of support groups felt confident about their potential to alleviate loneliness, there were also reports of ostracisation due to a lack of ‘fit’ within support group narratives. Thirty-two-year-old Natalie, who has CAIS, felt that her experiences had not been incorporated into online discussions of her variation because she had been diagnosed at twenty-one years old, which she understood to be relatively late, and had not received any surgical interventions, which she believed was unusual. Natalie explained:

You look at all the advice websites, there's … websites that give advice for parents of children who’ve got the condition, all the advice is aimed at people who’ve had treatment – the surgery done. But there's no advice for those who still haven’t had the surgery. And therefore, then I get frustrated when doctors turn around when I – they say to you ‘well there is a special support group’, it's useless. Because it doesn’t support somebody who's still got the problem. They support people who’ve, y’know, on the after-side of the procedure, not who's still going through it all, y’know so it's … I don’t bother with support groups.

Due to her atypical circumstances, Natalie described speaking in a Facebook support group as ‘like standing in a crowded room *screaming* and nobody's looking at you’. In agreement, Rosedale (2009) described the moral imperative survivors of breast cancer felt to withhold aspects of their experiences assumed to be unwelcome. She explained, ‘when faced with other survivors, they could silence or chasten themselves for thoughts they characterized as selfish or for an insufficiency of “fighting spirit”’ (Rosedale, 2009: 180). Consequently, particular narratives become dominant within certain spaces, discouraging those with differing experiences or feelings from sharing their perspectives. In these instances, Natalie felt that her attempts to seek advice and reassurance were undermined, and the exclusion of her experiences – especially within networks specifically designed for community support – only added to her feelings of isolation, leading her to eventually abandon the groups altogether.

Similarly, after years of searching for medical assistance without receiving any definite diagnosis, Ian spoke about the loneliness he felt due to a lack of peer support or medical label:

I still feel kind of … alone, in that: one, is that I never really got my tests confirmed, and two, that I never really proved to doctors that my symptoms exist. So, I still feel quite alone. I don’t really have any support. Well I have *online*, but it's more generic kind of thing but it's … I don’t really have anyone I can confide in.

Ian made a connection between the absence of a definite medical diagnosis or affirmation of his experiences, and the inadequate level of support he had received, resulting in loneliness. Due to the presumed ascendency of the medical institution and its monopoly over defining bodies, the lack of a confirmed diagnosis meant Ian had not received the (conditional) epistemological legitimacy largely imputed to medical labels (see: Parsons, 1975). Ian perceived this to impact his ability to understand and make sense of his experiences. His inability to achieve a definite response from medical authorities also meant there have been limited opportunities for him to participate in the construction of a collective identity or in a biosocial community (Rabinow, 1996).

Ian's difficulty achieving medical confirmation may also play a role in the sense of responsibility he felt for his own marginalisation, as diagnostic labels hold the potential to absolve individual accountability and judgment (Freidson, 1970: 248), and provide ‘tools to palliate and explain what makes him or her different’ (Jutel, 2009: 279). Whilst people with sex variations are often understood to ‘begin their lives in liminality’ (Hester, 2006: 46), a persistent sense of uncertainty and transitionality continued over time for Ian, as he felt acutely conscious of his body's resistance to fit others’ expectations, and had still not been given adequate medical recognition. An unaccepting and stigmatising society may have led Ian to seek a diagnosis – internalising the medical gaze (Foucault, 2003) – in an attempt to be purged of blame. However, Ian's persistent exclusion from diagnostic legitimacy illustrates the dangers of using the fluctuating benchmarks of sex classification to find acceptance (Griffiths, 2018), as ontological loneliness, too, emerged through his systemic elimination from the classification system.

### Difference

The self-perception of difference – as a form of abnormality and exceptionality – was widespread across participants with VSCs. The significance of this difference was often instilled by medical practitioners and parents from the earliest stages of diagnosis (and in some cases, prior to/without diagnosis). The rarity of their sex variations was underscored, and they often believed there was no one who shared their diagnosis. For example, as Paula, thirty-five years old, said: ‘my parents weren’t ever given the name [of the diagnosis of PAIS]. They were literally told we’d never find someone else in the country with the same condition as I had’. Paula's parents, like others in this research, were reported to frame their child's sex variation in a positive light: a characteristic which denotes ‘specialness’, rather than a problem. Paula recalled that her mother ‘always told me that I was special, I had no ovaries, couldn’t have babies, wouldn’t have periods, y’know – it was kind of the same spiel every time’. Siân, twenty-eight years old, described something similar, ‘I s’pose, when I was at primary school it was sort of – I saw [Turner syndrome] as something that was different, but it was – […] my parents sort of got me to sort of understand […] that it was something that was a bit special? – rather than sort of different being a *bad* thing’.

Some participants drew a connection between their feelings of difference and the imposition of secrecy. Steve, fifty-four years old with a diagnosis of PAIS, wrote in his diary, ‘I learned that i was different, that i had to live in a way that NO ONE was to know’. Steve's ascription of difference was externally stipulated. Similarly, Paula reflected that ‘it was drummed into us at a really early age, that we’d never find anyone with it, keep it quiet, it's quite shameful so keep shut up basically’. Both Steve and Paula connected the stringent constraints of secrecy and the singularity of VSCs to an instructed and learnt sense of shame and anxiety, a ‘desire to conceal oneself’ (Dolezal, 2015: 574). Steve explained, ‘I feared people knowing I was different. I feared the repercussions of people knowing I was different’. Steve's sense of deviation led to concerns about the outward visibility of his variation and an anticipation of hostile reactions if detected, further reproducing his feelings of self-scrutiny and singularity, resulting in ontological loneliness.

Pandora acknowledged the beneficial or tolerable aspects of some kinds of difference. However, she underlined divergence from the sex binary as a fundamental problem, casting her as singularly deviant:

I *want* to be different, but not in that, almost it feels like, such a crucial way. Or, it has done – of that … ‘humans are male or female’, and if I don’t … that is *the crucial difference*. And that's the one where if we’re going to fit in, the one thing that we need to be is ‘normal’, in that sense. […] And yeah, *categorisable*, I s’pose, in that way. For *myself* as much as for anyone else, just so that I wasn’t as – I wasn’t this anomaly.

Due to her experiences of shame, secrecy and renunciation, Pandora understood sex binarism to be an essential requirement for social recognition and belonging. Her realisation that ‘normal's just a silly idea’ potentially demonstrated an awareness of the regulating and ideological function of normalcy, but this did not provide any consolation while social and institutional responses continued to frame people's bodies with sex variations as ‘a “problem” in need of fixing’ (Hester, 2006: 48).

Drawing attention to the singularity or rarity of identities or morphologies inevitably alters the way that we conceptualise and respond to them. Indeed, as Feder (2014: 15) argues, the framing of intersex variations as ‘extraordinary’ cases, or ‘disorders like no other’ provides justification for the ‘routine violations of well-established ethical principles’. Individualising experiences also works to minimise the effects of, and move our attention away from, harmful medical and discursive processes. As Davis notes with regards to ableism, the individualisation of disabled people through their portrayal as ‘“noble,” “heroic,” and “special”’, is a denigrating and patronising ‘attempt to redress the disability by attributing higher powers to it’ (1995: 106). Whilst parents who affirm the status of their child with a VSC as ‘special’ may have benign intentions, this privileging of the ‘inherent powers’ of marginalised bodies or identities can also contribute to further separation and singularisation, and may increase their exposure to loneliness and isolation. Indeed, the depiction of Paula's variation as entirely unique initially deprived her of the possibility of finding a community or exchanging experiences, imposing a separation between her and the many others who shared her diagnosis.

In recent decades, we have seen the emergence of intersex political activism and advocacy initiatives, introducing structural critiques of medical interventions, with the fundamental goal ‘to change the way intersex infants are treated’ and ‘bodies are managed’ (Chase, 1998: 197–198). These arguments were born through the communities and groups formed around intersex and VSC experiences, intended for support with ‘shame, stigma, grief, and rage as well as with practical issues’ (Chase, 1998: 197). Just as we saw in the formative consciousness-raising of the women's liberation movement, collective traumas were acknowledged within these spaces, putting a spotlight on the ‘unspeakable’ (Fricker, 2007). The politicisation of the movement and the development of these institutional critiques also brought ‘clarity, cognitive confidence’ (Fricker, 2007: 148) and the opportunity to protest. These established communities, Delimata suggests, offer a space where ‘atypical sex is normal’ and ‘sex and identity are coherent’ (2019: 51). Until more fundamental social and structural change is achieved, these nascent sites of recognition may also begin to mitigate a sense of incoherence, hermeneutical marginalisation and the impact of loneliness.

### Conclusions

In the spirit of Mills ([1959] 1978), this article considers how a very personal and private experience of loneliness can be interpreted as a public, structural and social concern. Whilst loneliness is not an easily calculable or predictable feeling, some of the specific social repercussions of an intersex diagnosis – such as shame, stigma, rejection, enforced secrecy and the fundamental presumption of a ‘treatable’ difference – were recurring in participants’ accounts. These accounts illustrate how loneliness due to sex variations does not emerge organically; rather, it is produced as a result of specific systemic acts of abandonment, mistreatment and silencing. These traumas, and their state sanction and normalisation through the medicalisation of VSCs, created the conditions for ethical and ontological manifestations of loneliness. Stories of parenting illustrated how – when diagnoses occurred in infancy – social exclusion and abandonment were anticipated from the earliest possible moment. Echoing Garland and Travis (2020b: 26), I argue that intersex mistreatment and loneliness must be understood as ‘a structural and systemic issue that requires a state response’ and social change.

As participants with VSCs indicated, medical authorities’ claims that their diagnoses (and thus bodies) were especially rare – and that meeting others was infeasible – obstruct the possibility of finding a community. However, feelings of social exclusion and loneliness were also discussed as distinct from concerns about relationships and social connections. Exclusion described by participants can be interpreted as ontological, whereby hegemonic narratives of acceptable bodies and normative development produced a social environment in which participants did not feel valued, or felt they did not ‘fit’ or belong. This ontological loneliness was experienced by some participants with VSCs as a repudiation of their status, bodies and futures, placing limitations on ways of being. In some cases, this resulted in an imperative to receive surgical and hormonal interventions, and to secrecy regarding their VSC, treatments and medical history (even when surgeries were consensual or solicited).

Due to the perception that VSCs and their medicalisation made people's bodies and identities shameful, inadequate and undeserving, some participants described a self-imposed isolation, in which friendships and intimate relationships were avoided. At times, this led participants to believe they were responsible for their own suffering, despite experiences of unwarranted abuse and discrimination. Attempts to resist hegemonic constraints of ‘normal’ and acceptable identities, bodies and behaviour were perilous, due to the risk of further marginalisation if they were believed to be untrustworthy. The epistemological dominance of medical and social beliefs about binary sex very often leaves people diagnosed with VSCs without the confidence required to challenge their categorisation and treatment plan. The sense that there is no opportunity for negotiation or resistance (especially in diagnosis during infancy) can lead to a sense of powerlessness and an absence of jurisdiction over their bodies and the direction of their lives (Jones, 2016). Some highlighted the importance of support networks for finding acceptance, shared experiences and respite from isolation. In some instances, these networks were sites not just of sociality but of ontological security: a comfortable place to be who you are, and to find others who share and value that state of being. A complex interaction with medicalisation emerges here. For some, it was a shared diagnosis or sense of biosociality which initially made this possible, whilst for others their isolation was connected to the stigma of pathologisation, the narrow parameters of diagnostic criteria or the socially expected diagnosis/treatment trajectories.

The research process itself was situated by some participants as an opportunity for sharing unspoken experiences and feelings that were usually concealed due to both medical and social shaming. For some, this was a resistance to the imposition of silence and their first opportunity to grant someone else access to this private, hidden knowledge. Pandora, for example, explained that our encounter was of deep significance to her, providing a rare opportunity to acknowledge her sense of self in the company of someone sympathetic to her situation. Whilst the purpose of the research was not to provide emotional support to participants or to alleviate loneliness specifically, many of them alluded to the therapeutic benefits of taking part. The importance they placed on their involvement and the opportunity to speak freely is indicative of the inadequate social support currently available, and our collective ongoing failure to listen to these stories.
